# Thiazole and Oxazole Alkaloids: Isolation and Synthesis

**DOI:** 10.3390/md8112755

**Published:** 2010-11-05

**Authors:** Danilo Davyt, Gloria Serra

**Affiliations:** Cátedra de Química Farmacéutica, Facultad de Química, UdelaR, General Flores 2124, Montevideo, Uruguay

**Keywords:** thiazole, oxazole, marine

## Abstract

Thiazoles, oxazole and their corresponding reduced derivatives, thiazolines and oxazolines, are found in marine sources exhibiting significant biological activities. The isolation, synthetic, and biological studies of these natural products, covering literature from January 2007 to June 2010, are summarized.

## 1. Introduction

The role of natural products in drug discovery has undergone many changes over the past two decades, from a decline in participation by pharmaceutical companies in the 1990s, to a renaissance in recent years. In addition, the expectations from combinatorial libraries in drug screening have not been fulfilled and more than 60% of prescription drugs are of natural product origin. As a consequence, the natural product-inspired drug discovery and development has received renewed attention in recent years.

A large number of natural products, in particular from the marine environment, contain thiazole, oxazole, thiazolines or oxazolines heterocycles. In many cases, promising anti-tumor, antibacterial, anti-viral, anti-malaria and anthelmintic activities have been identified for these compounds. This review focusses on the isolation and structural determination, biological activities and synthetic studies of these marine products covering literature from January 2007 to June 2010.

## 2. New Thiazoles Isolated from Marine Sources

**Figure 1:**
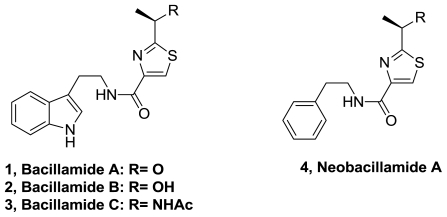
Structures of bacillamide A, B and C and neobacillamide A.

The known algaecide bacillamide A (**1**) and two new analogues, bacillamides B (**2**) and C (**3**), Figure 1, were isolated from *Bacillus endophyticus* obtained from a Bahamian hypersaline microbial mat [1]. The detection of these metabolites containing tryptamide thiazole motif, was performed using HPLC-UV-MS bioassay technique. Due to a lack of sufficient material and the appropriate test organisms, bacillamides B and C were not tested for algicidal activity. None of the purified bacillamides demonstrated antibiotic activity against target isolates of hypersaline pond *Bacillus* sp. at concentrations less than 500 μM.

A related compound, neobacillamide A (**4**), Figure 1, together with bacillamide C, was isolated from the bacterium *Bacillus vallismortis* C89 associated with the sponge *Dysidea avara* [2]. Neobacillamide A represents the first example of a thiazole-carboxamide bearing a 2-phenylethylamine moiety. Both compounds, Neobacillamide and Bacillamide C were inactive as cytotoxic against HL60 human leukemia cells and A549 human lung cancer cells.

**Figure 2:**
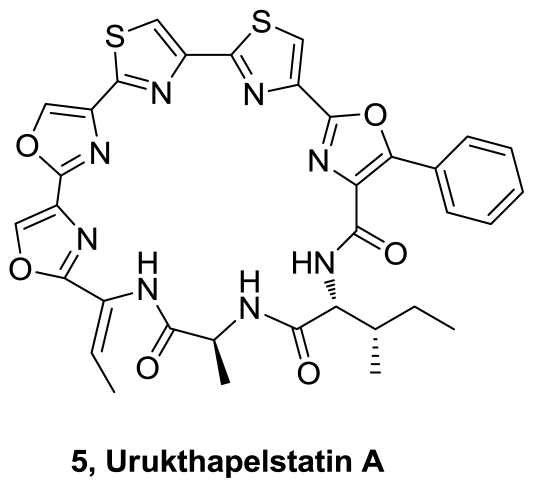
Structures of urukthapelstatin A.

A thiopeptide antibiotic, urukthapelstatin A (**5**), Figure 2, was isolated from a culture of *Thermoactinomycetaceae* bacterium *Mechercharimyces asporophorigenens* YM11-542 [3]. Its structure was determined by spectroscopic methods and chemical degradation, and confirmed by X-ray crystallographic analysis. It is related to 24 membered cyclic compounds mechercharstatin and telomestatin. Urukthapelstatin A inhibited the growth of human lung cancer A549 cells with an IC_50_ value of 12 nM and also showed potent cytotoxic activity against a human cancer cell line panel.

**Figure 3:**
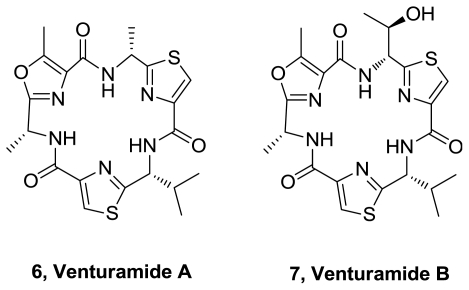
Structures of venturamide A and B.

Two new cyclic hexapeptides, venturamides A (**6**) and B (**7**), Figure 3, were isolated from the anamanian marine cyanobacterium *Oscillatoria* sp. [4]. Venturamide A and B showed *in* v*itro* antimalarial activity against *Plasmodium falciparum* (8.2 and 5.6 μM respectively), with only mild cytotoxicity to mammalian Vero cells (86 and 56 μM respectively). They exhibited only mild activity against *Trypanasoma cruzi* and *Leishmania donovani*.

**Figure 4:**
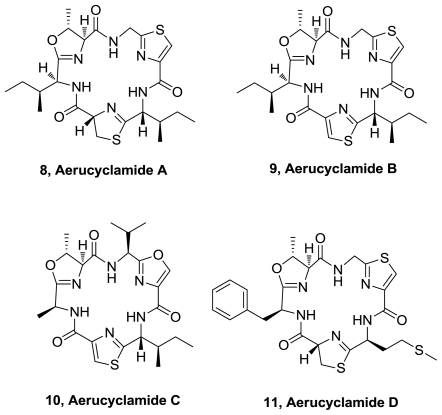
Structures of aerucyclamides A, B, C and D.

Four new modified hexacyclopeptides, aerucyclamides A (**8**), B (**9**), C (**10**) and D (**11**), Figure 4, were isolated from the toxic freshwater cyanobacterium *Microcystis aeruginosa* PCC 7806 [5,6]. These compounds encompass hexameric cyclopeptides alternating in hydrophobic aminoacids and oxazoline, thiazoline, thiazole or oxazole heterocycles derived from hydrophilic aminoacids (Ser, Thr, Cys). Aerucyclamide B displayed a submicromolar (0.7 μM) IC50 value against the chloroquine-resistant strain K1 of *P. falciparum*. In addition, this compound displays a large selectivity for the parasite with respect to the L6 rat myoblast cell line. Aerucyclamides A, C y D displayed low micromolar activity against *P. falciparum*. The most active compound against *T. brucei rhodesiense*, is aerucyclamide C (**10**), albeit with moderate activity.

**Figure 5:**
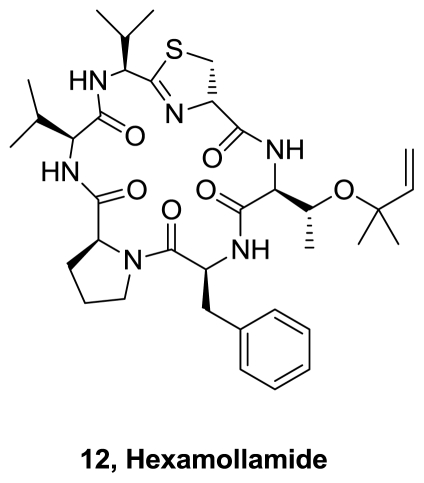
Structure of hexamollamide.

A new hexapeptide, Hexamollamide (**12**), Figure 5, was isolated from an Okinawan ascidian *Didemnum molle* [7]. The relative stereostructure was confirmed by X-ray crystallographic analysis. Hexamollamide contains a thiazoline ring and a threonine amino acid, which is modified by attachment to a dimethylallyl ether. It is structurally similar to patellin 2 isolated from *L. patella* [8,9]. Hexamollamide showed moderate cytotoxicity against HeLa S3 cells, with an IC_50_ value of 17 μg/mL.

**Figure 6:**
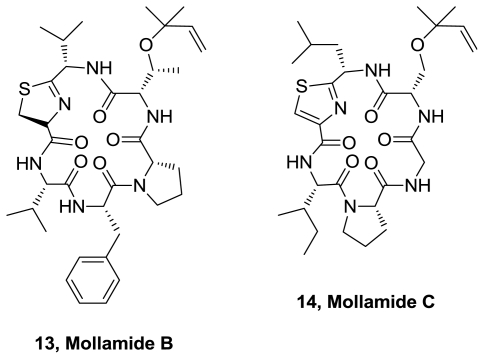
Structures of mollamide B and C.

Two new cyclic hexapeptides, mollamides B (**13**) and C (**14**), Figure 6, were isolated from *Didemnum molle* collected from Manado Bay [10]. They contain threonine or serine amino acids, modified by attachment to a dimethylallyl ether. The relative configuration of mollamide B at the thiazoline moiety was determined using molecular modeling coupled with NMR-derived restraints. Their absolute configuration was determined using Marfey’s method. Mollamide B (**13**) exhibited moderate antimalarial activity against *Plasmodium falciparum* (D6 clone and W2 clone), with IC_50_ values of 2.0 and 2.1 μg/mL, respectively. Mollamide B also exhibited marginal activity against *Leishmania donovani*, with IC_50_ and IC_90_ values of 18 and 35 μg/mL and cytotoxicity against several cancer cell lines. It showed neither antimicrobial nor anti-inflammatory activities. Mollamide C has not shown antiinflamatory activity and it was not considered to be solid tumor selective.

**Figure 7:**
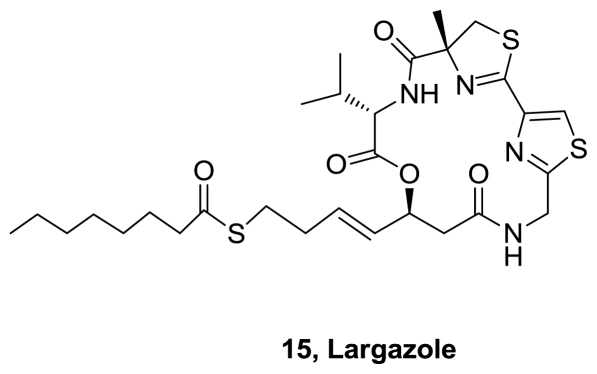
Structure of largazole.

In 2008, a new metabolite with novel chemical scaffold and nanomolar antiproliferative activity, largazole (**15**), Figure 7, was isolated from a sample of a cyanobacterium of the genus *Symploca* collected from Key Largo, Florida Keys, U.S. [11]. It potently inhibited the growth of highly invasive transformed human mammary epithelial cells (MDA-MB-231) (GI_50_ = 7.7 nM). In contrast, nontransformed murine mammary epithelial cells (NMuMG) were less susceptible (GI_50_ = 122 nM). The authors suggested that cancer cells are preferentially targeted by largazole. It possesses a combination of unusual structural features, including a substituted 4-methylthiazoline fused to a thiazole, a thioester moiety that has not been reported in metabolites from cyanobacteria and the unprecedented in marine natural products 3-hydroxy-7-mercaptohept-4-enoic acid unit.

**Figure 8:**
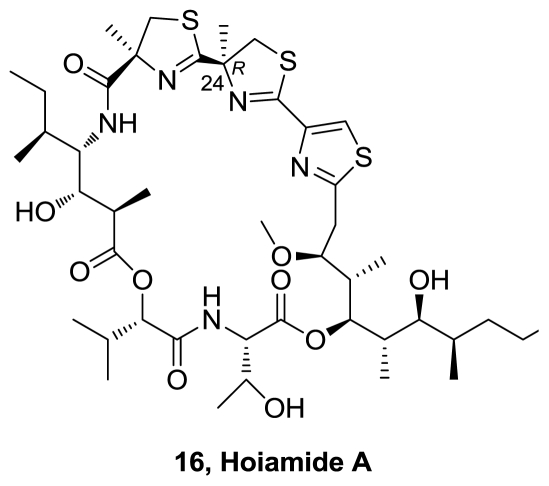
Structure of hoiamide A.

From an environmental assemblage of the marine cyanobacteria *Lyngbya majuscula* and *Phormidium gracile* collected in Papua New Guinea, a novel bioactive cyclic depsipeptide, hoiamide A (**16**), Figure 8, was isolated [12]. The structure was then corrected by the authors in an erratum, showing R configuration at C24 (instead of the S configuration pictured in the original paper) [13]. This metabolite possesses an unusual structure derived from a mixed peptide-polyketide biogenetic origin, and includes three fragments: a triheterocycle bearing two methylated thiazolines and one thiazol, a peptide and an oxygenated and methylated C15-polyketide. Hoiamide A exhibited modest cytotoxicity to cancer cells, but potently inhibited [^3^H]batrachotoxin binding to voltage-gated sodium channels (IC_50_ = 92.8 nM) and activated sodium influx (EC_50_ = 2.31 μM) in mouse neocortical neurons.

**Figure 9:**
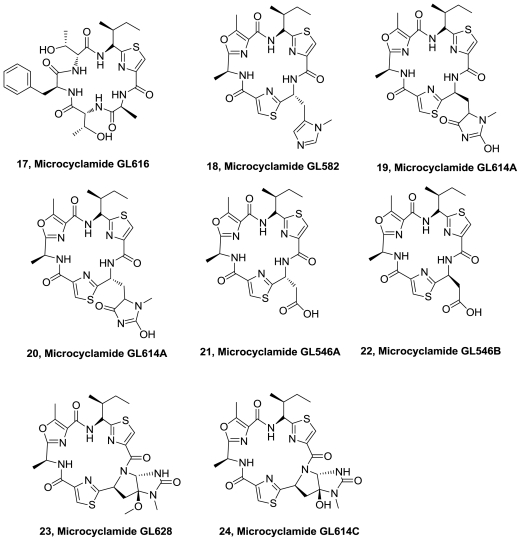
Structures of microcyclamides.

Eight novel thiazole and oxazole containing cyclic peptides, microcyclamides, GL616 (**17**), GL582 (**18**), GL614A (**19**), GL614B (**20**), GL546A (**21**), GL546B (**22**), GL628 (**23**), GL614C (**24**), Figure 9, were isolated from the hydrophilic extract of a *Mycrocystis* sp. water-bloom collected in Gelbea reservoir, Valley of Armagedon, Israel [14]. It is the first example where acidic and modified aminoacids are incorporated in this group of ribosomally biosynthesized metabolites. The compounds were assayed against one solid tumor (A549-lung) and one leukemis (Molt-4) cell lines. Microcyclamides GL582 (**18**) displayed weak cytotoxicity (20% grow inhibition) against Molt-4 cell line at 10 μM/mL.

## 3. Thiazolines Isolated from Marine Sources

**Figure 10:**
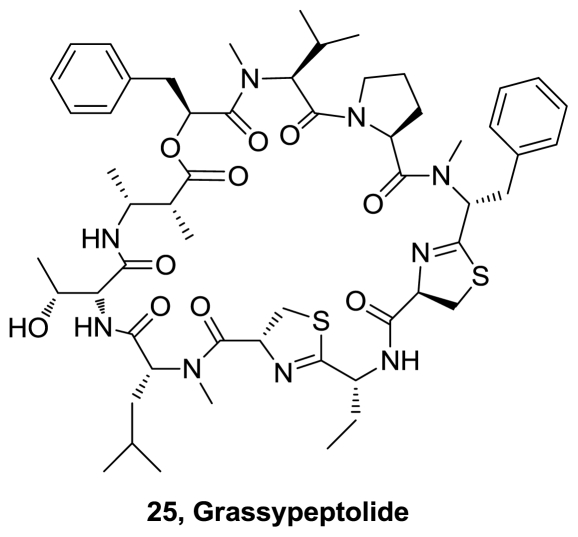
Structure of grassypeptolide.

A collection of the cyanobacterium *Lyngbya confervoides* off Grassy Key in Florida, U.S., yielded grassypeptolide (**25**), Figure 10, a 31-membered macrocyclic depsipeptide with unusually high d-amino acid content, two thiazolines, and one β-amino acid [15]. The structure was confirmed by X-ray crystallography. The antiproliferative activity was evaluated in four cell lines derived from human osteosarcoma (U2OS), cervical carcinoma (HeLa), colorectal adenocarcinoma (HT29), and neuroblastoma (IMR-32). Grassypeptolide showed moderate broad-spectrum activity with IC_50_ values of 2.2, 1.0, 1.5, and 4.2 μM, respectively.

**Figure 11:**
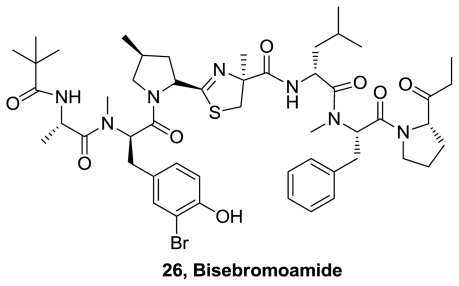
Proposed structure in the isolation report of bisebromoamide.

A new thiazoline metabolite, bisebromoamide (**26**), Figure 11, was isolated from the marine cyanobacterium *Lyngbya* sp. [16]. Its planar structure was determined by 1D and 2D NMR spectroscopy. The absolute stereostructure was determined by chemical degradation followed by chiral HPLC analysis. It contains a significant number of d-amino acids and *N*-methylated amino acids along with several other modified amino acid residues. Bisebromoamide exhibited antiproliferative activity at nanomolar levels and potent kinase inhibition.

## 4. Synthesis of Thiazoles of Marine Origin and Analogues

**Figure 12:**
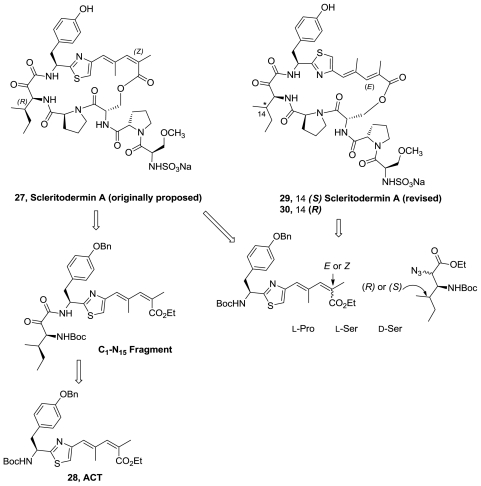
Retrosynthetic analysis of scleritodermin A.

The synthesis of the C1-N15 fragment of the originally proposed scleritodermin A (**27**), Figure 12, has been reported in good overall yield through a short and stereocontrolled sequence [17]. Highlights of the route include the synthesis of the novel ACT fragment (**28**) and the formation of the α-ketoamide linkage by the use of a highly activated α,β-ketonitrile.

In 2008, the first total synthesis of the originally proposed scleritodermin A (**27**) along with two isomers (**29**) and (**30**) were achieved [18]. The application of a transamination reaction was used for the formation of α-ketoamide. The authors reported that ^1^H and ^13^C NMR spectra of molecule **27** did not match those reported for natural scleritodermin. In contrast, the spectra of compound **29** and those reported for the isolated marine product were identical. Thus, compound **29** was proposed as the revised structure of scleritodermin A.

**Figure 13:**
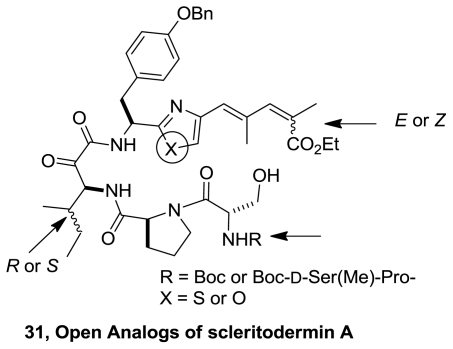
Synthesized analogs of scleritodermin A.

Recently, the synthesis of key fragments and open analogs of scleritodermin A and their biological evaluation as cytotoxic and anthelmintic were reported. Open and simplified analogs (**31**), Figure 13, were obtained in very good yield using a convergent strategy [19]. The compound with the highest antihelminthic activity is the open analog of **27** (LC_50_ = 9.6 μM). The presence, in some obtained compounds, of α,β-ketoamide function improves the cytotoxic activity on HCT-15 cells.

**Figure 14:**
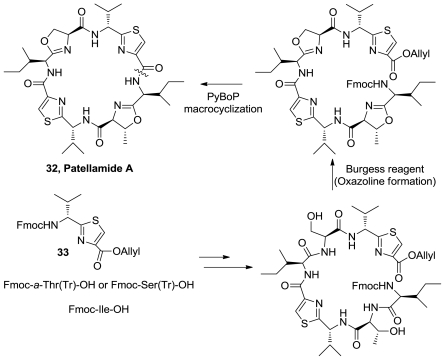
Synthetic route to patellamide A.

Pattellamide A (**32**), Figure 14, was synthesized from thiazole **33** and protected aminoacids using Burgess reagent to obtain the oxazolines rings. Macrocyclization was performed using PyBOP, DMAP, DIEA in CH_2_Cl_2_/DMF [20].

**Figure 15:**
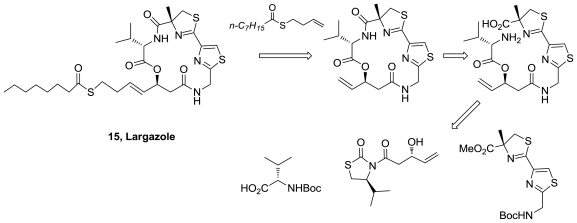
Retrosynthetic analysis of the first total synthetis of largazole.

The first total synthesis of Largazole (**15**), Figure 7, was reported four months after the publication of its isolation [21]. The synthesis (eight steps, 19% overall yield), involved a macrocyclization reaction for formation of the strained 16-membered depsipeptide core followed by an olefin cross-metathesis reaction for installation of the thioester, Figure 15. The biological evaluation of largazole and key analogues, suggested that histone deacetylases (HDACs) are molecular targets of largazole. The authors concluded that the cell growth inhibition is a functional consequence of I HDAC inhibition. In addition, structure-activity relationship (SAR) studies revealed that the thiol group is indispensable for both activities.

**Figure 16:**
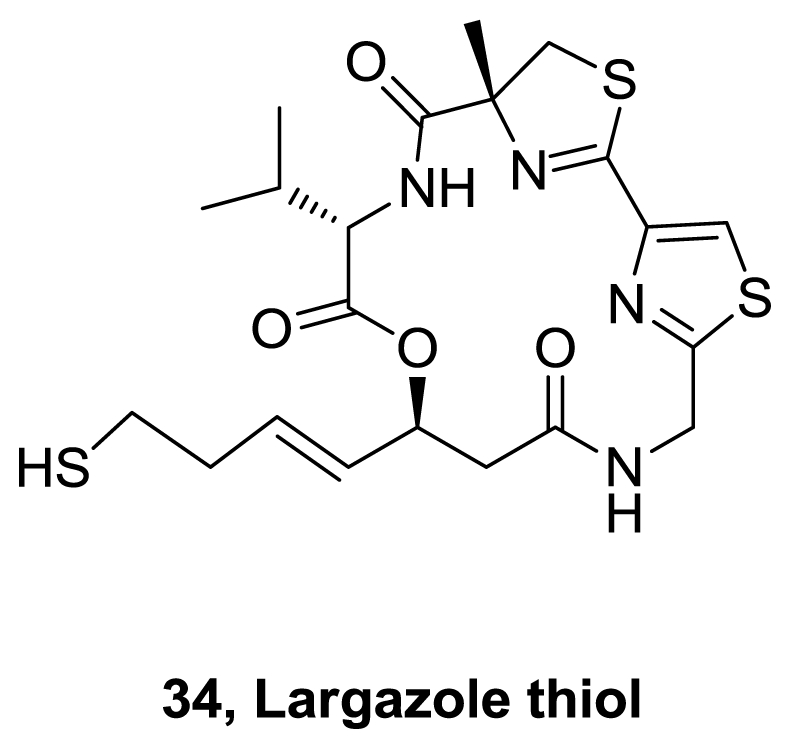
Active metabolite of largazole.

During 2008 seven more syntheses of largazole were reported [22–28]. Some of the used methodologies differ in the formation of the macrocycle or in the precursor of the 3-hydroxy-7-mercaptohept-4-enoic acid. In addition, some structure activity relationships studies were performed. It was demonstrated that largazole is a pro-drug that is activated by removal of the octanoyl residue from the 3-hydroxy-7-mercaptohept-4-enoic acid moiety to generate the active metabolite **34**, Figure 16, which is an extraordinarily potent Class I histone deacetylase inhibitor (*K_i_* = 0.07 nM) [22].

During 2009, six reports related to the synthesis of largazole and/or analogs were published [29–34]. A significant increase in potency with a pyridine substitution of the thiazole was reported. In addition, it was demonstrated that the methyl substituent of the thiazoline ring is nonessential for the dramatic potency of the natural product. In contrast, substitution of the thiazoline by thiazol, diminishes the activity [30].

Recently, new analogs of largazole were synthesized [35,36]. Structure-activity relationship studies suggested that the geometry of the alkene in the side chain is critical. While the largazole’s analogues with *trans*-alkene are potent for the antiproliferative effect, those with *cis*-alkene are completely inactive. In addition, replacement of valine by tyrosine increased selectivity toward human cancer cells over human normal cells more than 100-fold [35].

**Figure 17:**
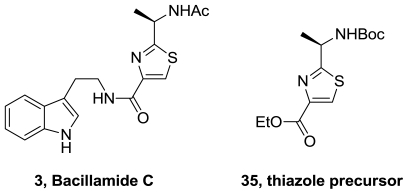
Thiazole precursor of bacillamide C.

Starting from d-alanine, bacillamide C (**3**), was obtained in 22% overall yield [37]. The key intermediate, thiazole **35**, Figure 17, was synthesized using Hantzsch reaction.

**Figure 18:**
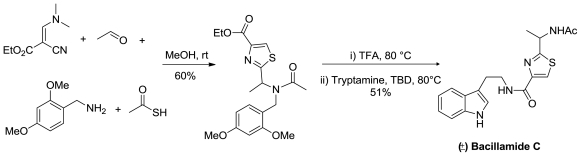
Synthesis of bacillamide C.

A thiazole Ugi multicomponent reaction and an aminolisis reaction were used to obtain racemic Bacillamide C, Figure 18 [38]. Additionally, several analogs of this natural product and a fluoro derivative of neobacillamide (**4**), Figure 1, were synthesized.

**Figure 19:**
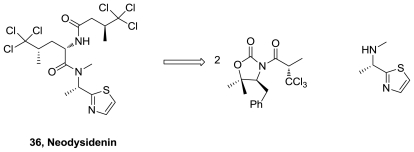
Retrosynthetic analysis of neodysidenin.

Recently, the enantioselective total synthesis of trichloroleucine-derived marine natural product neodysidenin (**36**), Figure 19, was accomplished. The strategy involves a direct ruthenium-catalyzed radical chloroalkylation capitalizing on the valence tautomerism of titanium enolates [39].

## 5. Synthesis of Thiazolines of Marine Origin and Analogues

**Figure 20:**
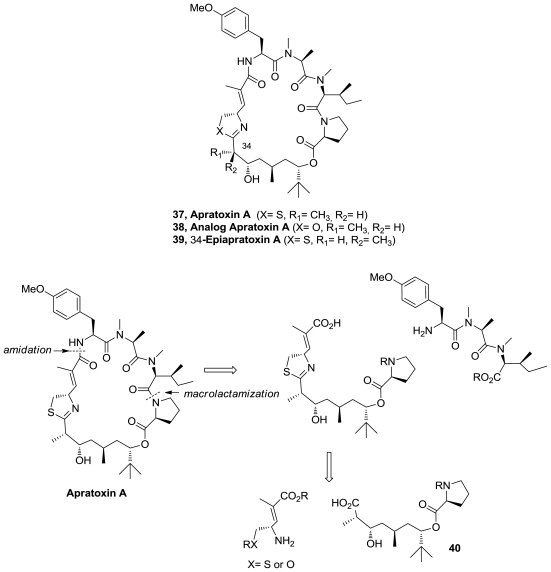
Apratoxin A analogs and retrosynthetic analysis of apratoxin A.

The cytotoxic thiazoline, apratoxin A (**37**), Figure 20, was synthesized in 18 steps and 18% overall yield. In addition, an oxazoline analogue (**38**) and 34-epiapratoxin A (**39**) were obtained [40]. The synthetic strategy to obtain apratoxin A involves the preparation of fragment **40** through a stereoselective route based in three asymmetric reactions. Thiazoline formation was successfully accomplished from a Cys amide using Ph_3_PO/Tf_2_O and macrolactamization was performed between *N*-methylisoleucine and proline residues using HATU.

**Figure 21:**
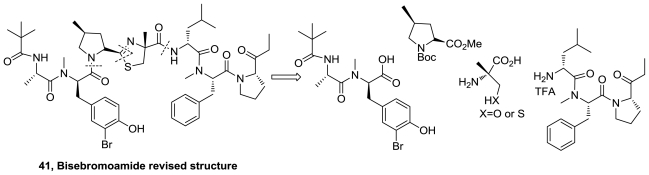
Retrosynthetic analysis of bisebromoamide.

Recently, the first total synthesis and a revised configurational assignment of bisebromoamide were reported [41]. The authors synthesized the originally proposed structure (**26**), Figure 11, but, neither NMR spectra nor optical rotation for the obtained compound were identical with those of the natural product. The authors elected to synthesize the epimer with *R*-configuration of the stereogenic center at the thiazoline ring of the proposed structure (**41**), Figure 21, from different fragments as is shown in the retrosynthetic analysis. The spectral data and optical rotation for **41** were identical to those of the natural product leading to a revision of the reported stereochemistry of bisebromoamide.

## 6. New Oxazoles Isolated from Marine Sources

**Figure 22:**
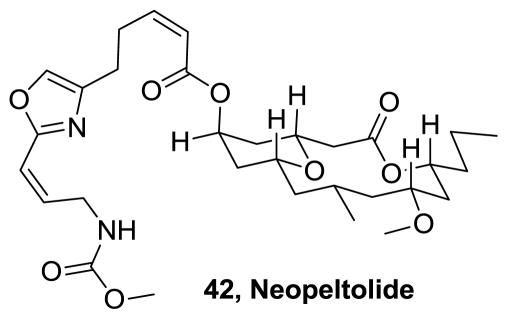
Structure of neopeltolide.

A new marine-derived macrolide designated as neopeltolide (**42**), Figure 22, was isolated from a deep-water sponge of the family *Neopeltidae*. Its structure was elucidated on the basis of spectroscopic data interpretation. Neopeltolide is a potent inhibitor of the *in vitro* proliferation of the A-549 human lung adenocarcinoma, the NCI-ADR-RES human ovarian sarcoma, and the P388 murine leukemia cell lines, with IC_50_ of 1.2, 5.1, and 0.56 nM, respectively. **42** also inhibited the growth of the fungal pathogen *Candida albicans* with a minimum inhibitory concentration of 0.62 μg/mL [42].

**Figure 23:**
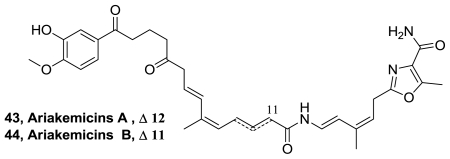
Structure of ariakemicins A and B.

Ariakemicins A (**43**) and B (**44**), Figure 23, unusual linear hybrid polyketide-nonribosomal peptide antibiotics, were discovered from the fermentation extract of the marine gliding bacterium *Rapidithrix* sp. [43]. These metabolites were positional isomers with regard to a double bond and chromatographically inseparable, rendering the structure study on a mixture basis. The antibiotics selectively inhibited the growth of Gram-positive bacteria.

**Figure 24:**
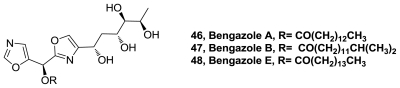
Structure of bengazoles A, B and E.

Bioassay-guided fractionation of extract of the sponge *Dorypleres splendens* has led to the discovery of a new natural source of bengazole A (**45**), B (**46**), and E (**47**), Figure 24, together with bengamide A. It had been thought that these compounds were confined to *Jaspis* species. This is the first time that bengazoles were isolated from another genus. Those compounds showed growth inhibitory activity to seven murine and human cancer cell lines [44].

**Figure 25:**
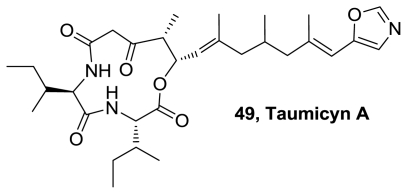
Structure of taumycin A.

An oxazole lipodepsipeptide, taumycins A (**49**), Figure 25, were isolated from the Madagascar sponge *Fascaplysinopsis* sp. with other closely related no oxazole containing metabolite taumycine B. The two compounds have the same 12-membered oxodepsipeptide ring system in common. Both were toxic to brine shrimp larvae, and taumycin A (1 μM), but not taumycin B, inhibited growth of the human UT-7 leukemic cell line. The structure of the two compounds, likely to be derived from microorganisms, was established by MS and 1D and 2D NMR data [45].

**Figure 26:**
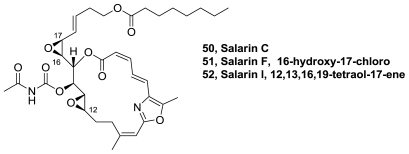
Structure of salarins C, F and I.

A novel oxazole macrolide, salarin C (**50**), Figure 26, was isolated from the Madagascar sponge *Fascaplysinopsis* sp. The structure of the compound was elucidated by interpretation of MS and 1D and 2D NMR spectra. Salarin C (**50**) is closely related to salarin A and is considered to be the precursor of salarins A and B. Air oxidation was found to transform **50** to salarin A. **50** was found to inhibit cell proliferation of human leukeamic cell lines UT-7 and K562, and the murine pro-B cell line Ba/F3 at concentrations of 0.0005–0.5 μg/mL [46].

Recently, seven new nitrogenous macrolides, designated salarins D-J, closely related to salarins A-C, were isolated from the same source. Salarin F (**51**) and I (**52**), Figure 26, have the same oxazole macrolide that **50**. All compounds were evaluated for their cytotoxicity against K562 and UT-7 human leukemia cells. While salarins D, E, H, and J displayed dose- and time-dependent inhibition of proliferation, **51** and **52** were not active in these assays [47].

**Figure 27:**
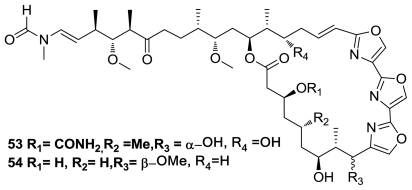
Structure of trisoxazole macrolides.

Two new trisoxazole macrolides, 9-*O*-desmethylkabiramide B (**53**) and 33-methyltetrahydrohalichondramide (**54**), Figure 27, were isolated in submicromolar amounts together with two new thiazole-containing cyclic peptides from a single specimen of *Hexabranchus sanguineus*, a nudibranch from the Indo-Pacific. The structure elucidation of these very minor metabolites was preformed using a 1 mm ^1^H NMR high-temperature superconducting microcryoprobe with unmatched sensitivity [48].

**Figure 28:**
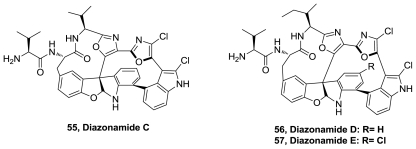
Structure of diazonamides.

Three new macrocyclic peptides, diazonamides C–E (**55**–**57**), Figure 28, were isolated together with the previously reported diazonamides A and B from samples of the marine ascidian *Diazona* sp. collected in Indonesia. The cytotoxic activity of the new compounds and diazonamide A was evaluated against a panel of three human tumor cell lines, including lung (A549), colon (HT29), and breast (MDA-MB-231). Moderate cytotoxicity with GI_50_ values in the micromolar range was found for the new compounds; whereas the potent activity previously reported for diazonamide A was confirmed with values in the low nanomolar range [49].

Enigmazole A (**58**), a novel phosphate-containing macrolide, was isolated from a Papua New Guinea collection of the marine sponge *Cinachyrella enigmatica* [50]. Compound **58** is comprised of an 18-membered phosphomacrolide that contains an embedded exomethylene-substituted tetrahydropyran ring and an acyclic portion that spans an oxazole moiety. Two additional analogues, 15-*O*-methylenigmazole A and 13-hydroxy-15-*O*-methylenigmazole A, were also isolated. The enigmazoles are the first phosphomacrolides from a marine source and **58** exhibited significant cytotoxicity in the NCI 60-cell line antitumor screen, with a mean GI_50_ of 1.7 μM.

## 7. Synthesis of Oxazoles of Marine Origin and Analogues

The relative stereochemistry of the metabolite (+)-neopeltolide (**42**), Figure 22, was originally proposed based on 1D and 2D NOESY spectra, which ultimately led to the incorrect structural assignment. The enantioselective total synthesis, with reassigned stereochemical and absolute configuration for neopeltolide (**59**), Figure 30, and their diastereomers has been reported almost simultaneously by Panek [51] and Scheidt [52].

Retrosynthetic strategy by Panek, Figure 31, began with disconnection of the C19–C20 double bond to reveal the macrolide **60** and the oxazole side chain **61**. Synthetic highlights of this route include a modified Evans-Tishchenko reduction to introduce the C11 stereocenter, [4 + 2] annulation to construct the pyran system, and a Still-Gennari olefination to install the oxazole side chain. Macrocyclization was performed through Yamaguchi esterification of the seco acid intermediate to form macrolide **60** in 44% yield. The longest linear sequence required 19 steps with an overall yield of 1.3%.

Scheidt and co-workers performed a macrocyclization of the linear precursor **62**, Figure 32, constructed from dioxinone acid **63** and alcohol **64**. In this key step, scandium-(III) triflate promoted the macrocyclization of **62** in a Prins type transformation. The synthetic neopeltolide inhibited proliferation of the murine leukemia line P388 with an IC_50_ of 0.6 nM and the human breast adenocarcinoma line MCF-7 with an IC_50_ value of 2.2 nM. Validating the revised structure as the correct one; diastereomer which corresponded to the originally proposed structure, was approximately 100-fold less active in both cell lines [53].

Five total syntheses of **59** have been reported in 2008 [54–58] and they were reviewed by Sasaki [59]. Since then, another four total syntheses were reported [60–64].

**Figure 29:**
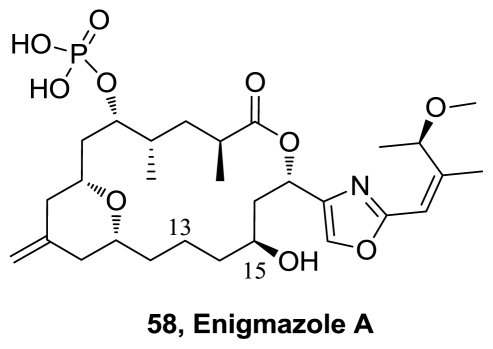
Structure of enigmazole A.

**Figure 30:**
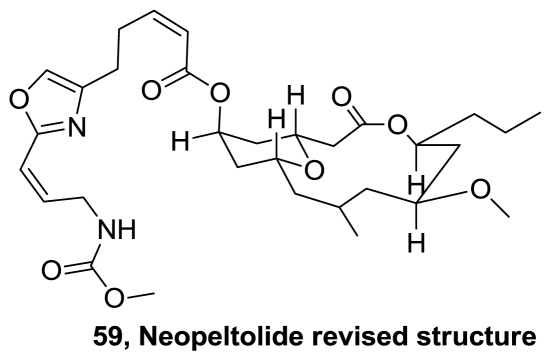
Revised structure of neopeltolide.

**Figure 31:**
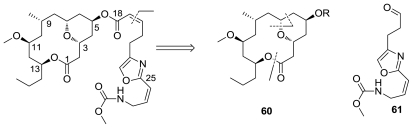
Retrosynthetic analysis of neopeltolide by Panek.

**Figure 32:**
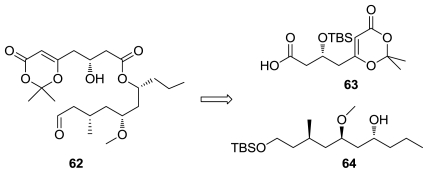
Retrosynthetic analysis of neopeltolide by Scheidt group.

**Figure 33:**
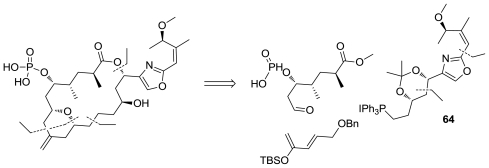
Retrosynthetic analysis of enigmazole A by Molinski.

The first total synthesis of the cytotoxic marine macrolide enigmazole A (**58**), Figure 29, was completed in 22 steps (longest linear sequence). The sensitive, densely functionalized 2,4-disubstituted oxazole fragment (**64**), Figure 33, was constructed using a Negishi-type coupling of an oxazol-2-ylzinc reagent formed directly from the parent ethyl 2-iodooxazole-4-carboxylate by zinc insertion. Other key steps include a hetero-Diels-Alder cycloaddition to form the central embedded pyran ring, a Wittig reaction to unite the hemispheres, and a ring size-selective Keck macrolactonization [65].

**Figure 34:**
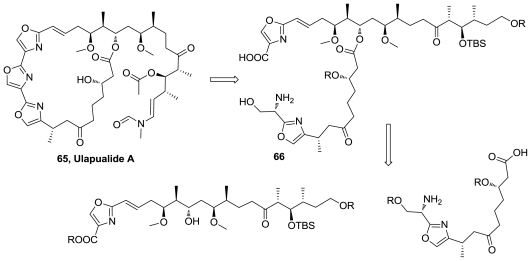
Structure of ulapualide A and their retrosynthetic analysis.

Stereochemistry of (−)-ulapualide A (**65**), Figure 34, was revised by X-ray analysis of its complex with the protein actin. A new total synthesis of **65** was reported, the first to yield a product that corresponds with the natural product. The synthesis involved the macrolactamization of the intermediate **66**, followed by elaboration of the central oxazole ring in **65** as a late step in the overall synthesis [66,67].

**Figure 35:**
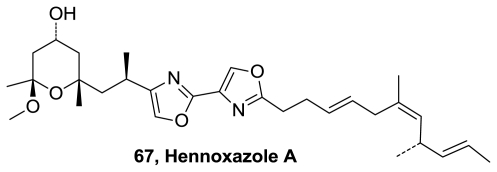
Structure of hennoxazole A.

An enantioselective, convergent, total synthesis of the antiviral marine natural product (−)-hennoxazole A (**67**), Figure 35, was completed in 17 steps, longest linear sequence, from serine methyl ester and in nine steps from an achiral bis-oxazole intermediate. Elaboration of a thiazolidinethione allowed for rapid assembly of the pyran-based ring system. Key late-stage coupling was effected by deprotonation of the bisoxazole methyl group, followed by alkylation with an allylic bromide side chain segment [68,69].

**Figure 36:**
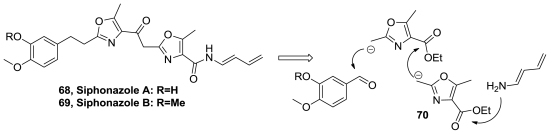
Structure of siphonazoles A–B and their retrosynthetic analysis by Ciufoloni group.

Siphonazoles A (**68**) and B (**69**), Figure 36, are structurally novel natural products isolated from a *Herpetosiphon species* [70]. They are the first, and so far the only, naturally occurring substances known that incorporate oxazole subunits connected by a two-carbon tether.

The first synthesis of a siphonazole was performed by Moody and Linder [71,72]. Key aspects of this work are the assembly of oxazole units via the reaction of rhodium carbenes with an amide or a nitrile, and the construction of the central (2-oxazolyl)methyl ketone motif by acylation of an organozinc agent derived from 4-carbomethoxy-2-iodomethyl-5-methyloxazole.

Other synthesis of siphonazoles was performed by Ciufoloni and co-workers involving the iterative use of a conjunctive oxazole building block that acts as a carrier of synthon **70**, Figure 36. Synthetic siphonazoles were devoid of antimicrobial activity, but they showed appreciable and selective cytotoxicity against human breast carcinoma HTB-129 (IC_50_ of 20 μg/mL) and acute T-cell leukemia TIB-152 (IC_50_ value equal to 16 μg/mL), with **68** being significantly more active than **69** [73].

The bengazoles are a family of metabolites isolated from a *Jaspis* sponge that display potent antifungal and anthelmintic activity, and have a unique structure containing two oxazole rings flanking a single carbon atom. Three new total synthesis of bengazoles [74–76] and analogs [77–79] were reported.

**Figure 37:**
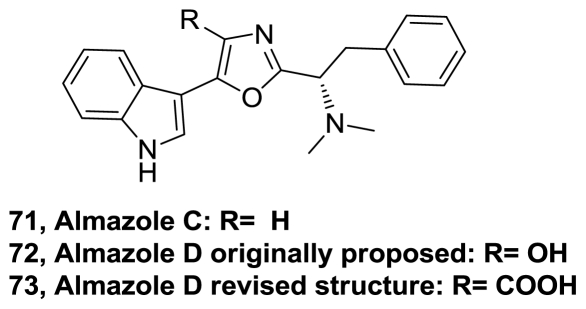
Structure of almazoles.

The marine alkaloid almazole C (**71**), Figure 37, isolated from the red seaweed *Haraldiophylum* sp. was synthesized in four steps. The key step, construction of the central 2,5-disubstituted oxazole ring, was based on the aza-Wittig reaction of the iminophosphorane derived from the α-azidoacetyl indole and (*S*)-*N*-phthaloylphenylalanyl chloride [80].

The structure of Almazole D (**72**), Figure 37, antibacterial metabolite isolated from a red seaweed from the Dakar coast [81], was revised by Horne and co-workers to **73**. Synthesis of **73** was performed by coupling the oxotryptophan methyl ester with *N*,*N*-dimethyl-L-phenylalanine and subsequent treatment with POCl_3_ [82].

**Figure 38:**
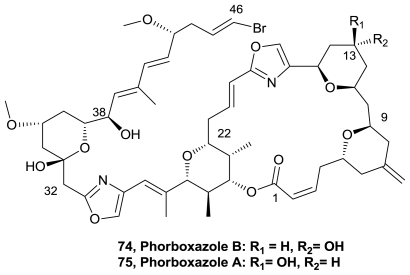
Structure of phorboxazoles.

The complex structure of phorboxazoles and their unique biological activity is a driving force for continued interest from the synthetic community. The phorboxazoles are among the most potent discovered cytostatic agents, exhibiting a mean GI_50_ < 1.58 × 10^−9^ M against the NCI panel of 60 tumor cell lines. Phorboxazole B (**74**), Figure 38, was prepared in 55 steps (longest linear sequence 28 steps) using a stereoselective hetero-Diels-Alder reaction to construct the key C33–C39 segment. The stereoselective hetero-Diels-Alder reaction of (*R*)-2,3-dibenzyloxypropanal and the Brassard diene EtO(Me_3_SiO)C:CHC(OMe):CH_2_ in presence of Eu(fod)_3_ provided dihydropyranone I, enabling the synthesis of the C18–C46 fragment of phorboxazole B. Coupling of the C18–C46 fragment with a suitable C3–C17 partner, followed by the late-stage formation of the C16–C18 oxazole unit and stereoselective macrocyclization and deprotection, rendered synthetic phorboxazole B [83].

Highly convergent syntheses of eight phorboxazole congeners and their evaluation against a diverse panel of human solid tumor cancer cell lines have been achieved. Specifically, the C(45–46) alkyne, alkene, and alkane phorboxazole A analogs were constructed and found to display single digit nanomolar cell growth inhibitory activities in a series of human cancer cell lines. The structurally simplified C(11–15)-acetal congener also proved potent, albeit reduced, when evaluated against the same cell line panel. Importantly, (+)-C(46)-chlorophorboxazole A displayed picomolar (pM) inhibitory activity in several cell lines [84].

A highly convergent second-generation synthesis of (+)-phorboxazole A (**75**), Figure 38, was achieved. Highlights of the synthetic strategy include improved Petasis-Ferrier union/rearrangement conditions on a scale to assemble multigram quantities of the C(11–15) and C(22–26) *cis*-tetrahydropyrans inscribed with the phorboxazole architecture, a convenient method to prepare *E* and *Z*-vinyl bromides from TMS-protected alkynes utilizing radical isomerization of *Z*-vinylsilanes, and a convergent late-stage Stille union to couple a fully elaborated C(1–28) macrocyclic iodide with a C(29–46) oxazole stannane side chain, to establish the complete phorboxazole skeleton. The synthesis, achieved with a longest linear sequence of 24 steps, resulted in 4.6% overall yield [85].

## 8. Synthesis of Oxazolines of Marine Origin and Analogues

**Figure 39:**
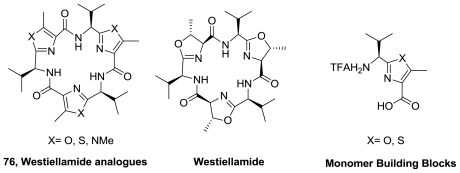
Westiellamide analogs and monomers building blocks.

The synthesis of westiellamide analogues of type **76**, Figure 39, wherein the oxazoline units were replaced by oxazole or thiazole units, was reported [86]. The strategy involved a one-pot macrocyclization of the monomer building blocks using diphenyl phosphorazidate (DPPA) in the presence of an excess of Hünig’s base under high dilution conditions at room temperature. The trimers **76** where X = O or S were obtained in 35% and 25% yield, respectively. The structures of these analogues, and also of an imidazole-based analogue, were investigated using X-ray diffraction and DFT-based molecular modeling calculations. The flexibility of the systems essentially depends on the type of the azole building block. The oxazole-based peptide is almost coplanar, whereas in the case of the thiazole and the imidazole cyclicpeptides the azole moieties form cone-like structures. Then, the copper(II) coordination chemistry of westiellamide, as well as of the three synthetic analogues (**76**) with an [18]azacrown-6 macrocyclic structure was reported [87]. The authors emphasized that, as in the larger patellamide rings, the *N*-heterocycle-*N*-peptide-*N*-heterocycle binding site is highly preorganized for copper(II) coordination. The macrocyclic peptides have been found to form stable mono- and dinuclear copper(II) complexes.

## References

[1] Aaron MS Richard AL David CR Bacillamides from a hypersaline microbial mat bacterium J Nat Prod 2007 70 1793 1795 17988095 10.1021/np070126a

[2] Yu L Li Z Peng C Li Z Guo Y Neoobacillamide A, a novel thiazole-containing alkaloid from the marine bacterium bacillus vallismortis C89, associated with south china sea sponge *Dysidea avara* Helv Chim 2009 92 607 612

[3] Matsuo Y Kanoh K Imagawa H Adachi K Nishizawa M Shizuri Y Urukthapelstatin A, a novel cytotoxic substance from marine-derived *Mechercharimyces asporophorigenens* YM11-542 J Antib 2007 60 256 260 10.1038/ja.2007.31 17456976

[4] Linington RG González J Urena L Romero LI Ortega-Barría E Gerwick WH Venturamides A and B: antimalarial constituents of the panamanian marine cyanobacterium *Oscillatoria* sp J Nat Prod 2007 70 397 401 17328572 10.1021/np0605790

[5] Portmann C Blom JF Gademann K Jüttner F Aerucyclamides A and B: isolation and synthesis of toxic ribosomal heterocyclic peptides from the cyanobacterium *Microcystis aeruginosa* PCC 7806 J. Nat. Prod 2008 71 1193 1196 18558743 10.1021/np800118g

[6] Portmann C Blom JF Kaiser M Brun R Jüttner F Gademann K Isolation of Aerucyclamides C and D and structure revision of Microcyclamide 7806A: heterocyclic ribosomal peptides from *Microcystis aeruginosa* PCC 7806 and their antiparasite evaluation J Nat Prod 2008 71 1891 1896 10.1021/np800409z 18973386

[7] Teruya T Sasaki H Suenaga K Hexamollamide, a hexapeptide from an Okinawan ascidian *Didemnum molle* Tetraedron Lett 2008 49 5297 5299

[8] Zabriskie TM Foster MP Stout TJ Clardy J Ireland CM Studies on the solution- and solid-state structure of patellin 2 J Am Chem Soc 1990 112 8080 8084

[9] Carroll AR Coll JC Bourne DJ MacLeod JK Ireland CM Bowden BF Patellins 1–6 and trunkamide A: novel cyclic hexa-, hepta- and octa-peptides from colonial ascidians, *Lissoclinum* sp Aust J Chem 1996 49 659 667

[10] Donia MS Wang B Dunbar DC Desai PV Patny A Avery M Hamann MT Mollamides B and C, Cyclic Hexapeptides from the Indonesian Tunicate *Didemnum molle* J Nat Prod 2008 71 941 945 18543965 10.1021/np700718p PMC2651694

[11] Taori K Paul VJ Luesch H Structure and activity of largazole, a potent antiproliferative agent from the floridian marine cyanobacterium *Symploca sp* J Am Chem Soc 2008 130 1806 1807 18205365 10.1021/ja7110064

[12] Pereira A Cao Z Murray TF Gerwick WH Hoiamide A, a sodium channel activator of unusual architecture from a consortium of two papua new guinea cyanobacteria Chem Biol 2009 16 893 906 19716479 10.1016/j.chembiol.2009.06.012 PMC2763540

[13] Pereira A Cao Z Murray TF Gerwick WH Hoiamide A, a sodium channel activator of unusual architecture from a consortium of two papua new guinea cyanobacteria Chem Biol 2009 16 1208 10.1016/j.chembiol.2009.06.012 PMC2763540 19716479

[14] Raveh A Moshe S Evron Z Flescher E Carmeli S Novel thiazole and oxazole containing cyclic hexapeptides from a waterbloom of the cyanobacterium *Microcystis* sp Tetrahedron 2010 66 2705 2712

[15] Kwan J Rocca JR Abboud KA Paul VJ Luesch H Total structure determination of grassypeptolide, a new marine cyanobacterial cytotoxin Org Lett 2008 10 789 792 18220404 10.1021/ol702946d

[16] Teruya T Sasaki H Fukazawa H Suenaga K Bisebromoamide, a potent cytotoxic peptide from the marine cyanobacterium *Lyngbya* sp. Isolation, stereostructure, and biological activity Org Lett 2009 11 5062 5065 19803465 10.1021/ol9020546

[17] Sellanes D Manta E Serra G Toward the total synthesis of scleritodermin A: preparation of the C1-N15 fragment Tetrahedron Lett 2007 48 1827 1830 18320006 10.1016/j.tetlet.2007.01.034 PMC1832122

[18] Liu S Cui Y-M Nan F-J Total synthesis of the originally proposed and revised structures of scleritodermin A Org Lett 2008 10 3765 3768 18686965 10.1021/ol801419m

[19] Sellanes D Campot F Nunez I Lin G Esposito P Dematteis S Saldana J Dominguez L Manta E Serra G Preparation and biological evaluation of key fragments and open analogs of scleritodermin A Tetrahedron 2010 66 5384 5395

[20] Garcia-Reynaga P VanNieuwenhze MS A new total synthesis of patellamide A Org Lett 2008 10 4621 4623 18808124 10.1021/ol801895y PMC4096709

[21] Ying Y Taori K Kim H Hong J Luesch H Total Synthesis and Molecular Target of Largazole, a Histone Deacetylase Inhibitor J Am Chem Soc 2008 130 8455 8459 18507379 10.1021/ja8013727

[22] Nasveschuk CG Ungermannova D Liu X Phillips AJ A Concise Total Synthesis of Largazole, Solution Structure, and Some Preliminary Structure Activity Relationships Org Lett 2008 10 3595 3598 18616341 10.1021/ol8013478 PMC2664405

[23] Seiser T Kamena F Cramer N Synthesis and biological activity of largazole and derivatives Angew Chem Int Ed 2008 47 6483 6485 10.1002/anie.200802043 18633950

[24] Bowers A West N Taunton J Schreiber SL Bradner JE Williams RM Total synthesis and biological mode of action of largazole: A potent class I histone deacetylase inhibitor J Am Chem Soc 2008 130 11219 11222 18642817 10.1021/ja8033763 PMC3090445

[25] Ghosh AK Kulkarni S Enantioselective total synthesis of (+)-largazole, a potent inhibitor of histone deacetylase Org Lett 2008 10 3907 3909 18662003 10.1021/ol8014623 PMC2945909

[26] Ying Y Liu Y Byeon SR Kim H Luesch H Hong J Synthesis and activity of largazole analogues with linker and macrocycle modification Org Lett 2008 10 4021 4024 18707106 10.1021/ol801532s

[27] Ren Q Dai L Zhang H Tan W Xu Z Ye T Total synthesis of largazole Synlett 2008 2379 2383

[28] Numajiri Y Takahashi T Takagi M Shin-ya K Doi T Total synthesis of largazole and its biological evaluation Synlett 2008 2483 2486

[29] Bowers AA Greshock TJ West N Estiu G Schreiber SL Wiest O Williams RM Bradner JE Synthesis and conformation-activity relationships of the peptide isosteres of FK228 and largazole J Am Chem Soc 2009 131 2900 2905 19193120 10.1021/ja807772w PMC2880701

[30] Bowers AA West N Newkirk TL Troutman-Youngman AE Schreiber SL Wiest O Bradner JE Williams RM Synthesis and histone deacetylase inhibitory activity of largazole analogs: alteration of the zinc-binding domain and macrocyclic scaffold Org Lett 2009 11 1301 1304 19239241 10.1021/ol900078k PMC2673910

[31] Seiser T Cramer N Syntheses and biological activity of the HDAC class I inhibitor largazole Chimia 2009 63 19 22

[32] Chen F Gao A-H Li J Nan F-J Synthesis and biological evaluation of C7-demethyl largazole analogues ChemMedChem 2009 4 1269 1272 19431162 10.1002/cmdc.200900125

[33] Yan W O’Doherty GA Total synthesis of (+)-largazole, a histone deacetylase inhibitor Chemtracts 2009 22 50 58

[34] Wang B Forsyth CJ Total synthesis of largazole-devolution of a novel synthetic strategy Syntesis 2009 2873 2880

[35] Zeng X Yin B Hu Z Liao C Liu J Li S Li Z Nicklaus MC Zhou G Jiang S Total synthesis and biological evaluation of largazole and derivatives with promising selectivity for cancers cells Org Lett 2010 12 1368 1371 20184338 10.1021/ol100308a PMC7386434

[36] Souto JA Vaz E Lepore I Poppler A-C Franci G Alvarez R Altucci L de Lera AR Synthesis and biological characterization of the histone deacetylase inhibitor largazole and C7-modified analogues J Med Chem 2010 53 4654 4667 20491440 10.1021/jm100244y

[37] Li D Yang HS Cui Q Mao SJ Xu XH Synthesis of bacillamide 3 and its analogue Chin Chem Lett 2009 20 1195 1197

[38] Wang W Joyner S Khoury KAS Doemling A (−)-Bacillamide C: the convergent approach Org Biom Chem 2010 8 529 532 10.1039/b918214d 20090966

[39] Beaumont S Ilardi EA Monroe LR Zakarian A Valence tautomerism in titanium enolates: catalytic radical haloalkylation and application in the total synthesis of neodysidenin J Am Chem Soc 2010 132 1482 1483 20078122 10.1021/ja910154f PMC2819066

[40] Numajiri Y Takahashi T Doi T Total synthesis of (−)-apratoxin A, 34-epimer, and its oxazoline analogue Chem Asian J 2009 4 111 125 19034894 10.1002/asia.200800365

[41] Gao X Liu Y Kwong S Xu Z Ye T Total synthesis and stereochemical reassignment of bisebromoamide Org Lett 2010 12 3018 3021 20527927 10.1021/ol101021v

[42] Wright AE Cook Botelho J Guzmán E Harmody D Linley P McCarthy PJ Pitts TP Pomponi SA Reed JK Neopeltolide, a macrolide from a lithistid sponge of the family Neopeltidae J Nat Prod 2007 70 412 416 17309301 10.1021/np060597h

[43] Oku N Adachi K Matsuda S Kasai H Takatsuki A Shizuri Y Ariakemicins A and B, novel polyketide-peptide Antibiotics from a marine gliding bacterium of the genus Rapidithrix Org Lett 2008 10 2481 2484 10.1021/ol8007292 18498148

[44] Pettit GR Hogan F Xu JP Tan R Nogawa T Cichacz Z Pettit RK Du J Ye QH Cragg GM Herald CL Hoard MS Goswami A Searcy J Tackett L Doubek DL Williams L Hooper JN Schmidt JM Chapuis JC Tackett DN Craciunescu F Antineoplastic Agents. 536. New Sources of Naturally Occurring Cancer Cell Growth Inhibitors from Marine Organisms, Terrestrial Plants, and Microorganisms J Nat Prod 2008 71 438 444 18327911 10.1021/np700738k

[45] Bishara A Rudi A Aknin M Neumann D Ben-Califa N Kashman Y Taumycins A and B, Two Bioactive Lipodepsipeptides from the Madagascar Sponge *Fascaplysinopsis* sp Org Lett 2008 10 4307 4309 18781810 10.1021/ol801750y

[46] Bishara A Rudi A Aknin M Neumann D Ben-Califa N Kashman Y Salarin C, a new cytotoxic sponge-derived nitrogenous macrolide Tetrahedron Lett 2008 49 4355 4358

[47] Bishara A Rudi A Aknin M Neumann D Ben-Califa N Kashman Y Salarins D–J, seven new nitrogenous macrolides from the madagascar sponge *Fascaplysinopsis* sp Tetrahedron 2010 66 4339 4345

[48] Dalisay DS Rogers EW Edison AS Molinski TF Structure elucidation at the nanomole scale. 1. Trisoxazole macrolides and thiazole-containing cyclic peptides from the nudibranch *Hexabranchus sanguineus *J Nat Prod 2009 72 732 738 19254038 10.1021/np8007649 PMC2753413

[49] Fernández R Martín MJ Rodríguez-Acebes R Reyes F Francesch A Cuevas C Diazonamides C–E, new cytotoxic metabolites from the ascidian *Diazona* sp Tetrahedron Lett 2008 49 2283 2285

[50] Oku N Takada K Fuller RW Wilson JA Peach ML Pannell LK McMahon JB Gustafson KR Isolation, Structural Elucidation, and Absolute Stereochemistry of Enigmazole A, a Cytotoxic Phosphomacrolide from the Papua New Guinea Marine Sponge *Cinachyrella enigmatica* J Am Chem Soc 2010 132 10278 10285 20590096 10.1021/ja1016766 PMC3850515

[51] Youngsaye W Lowe JT Pohlki F Ralifo P Panek JS Total Synthesis and Stereochemical Reassignment of (+)-Neopeltolide Angew Chem Int Ed 2007 46 9211 9214 10.1002/anie.200704122 18022886

[52] Custar DW Zabawa TP Scheidt KA Total Synthesis and Structural Revision of the Marine Macrolide Neopeltolide J Am Chem Soc 2008 130 804 805 18161979 10.1021/ja710080q

[53] Custar DW Zabawa TP Hines J Crews CM Scheidt KA Total synthesis and structure-activity investigation of the marine natural product neopeltolide J Am Chem Soc 2009 131 12406 12414 19663512 10.1021/ja904604x PMC2735232

[54] Woo SK Kwon MS Lee E Total synthesis of (+)-neopeltolide by a Prins macrocyclization Angew Chem Int Ed 2008 47 3242 3244 10.1002/anie.200800386 18348147

[55] Fuwa H Naito S Goto T Sasaki M Total synthesis of (+)-neopeltolide Angew Chem Int Ed 2008 47 4737 4739 10.1002/anie.200801399 18491342

[56] Paterson I Miller NA Total synthesis of the marine macrolide (+)-neopeltolide Chem Comm 2008 39 4708 4710 18830467 10.1039/b812914b

[57] Vintonyak VV Kunze B Sasse F Maier ME Total synthesis and biological activity of neopeltolide and analogues Chem Eur J 2008 14 11132 11140 10.1002/chem.200801398 18979467

[58] Kartika R Gruffi TR Taylor RE Concise Enantioselective Total Synthesis of Neopeltolide Macrolactone Highlighted by Ether Transfer Org Lett 2008 10 5047 5050 18855401 10.1021/ol802254z

[59] Sasaki M Fuwa H Total synthesis of (+)-neopeltolide, antitumor marine macrolide Farumashia 2009 45 439 443

[60] Guinchard X Roulland E Total Synthesis of the Antiproliferative Macrolide (+)-Neopeltolide Org Lett 2009 11 4700 4703 19775106 10.1021/ol902047z

[61] Fuwa H Saito A Naito S Konoki K Yotsu-Yamashita M Sasaki M Total Synthesis and Biological Evaluation of (+)-Neopeltolide and Its Analogues Chem Eur J 2009 15 12807 12818 10.1002/chem.200901675 19882704

[62] Fuwa H Saito A Sasaki M A Concise Total Synthesis of (+)-Neopeltolide Angew Chem Int Ed 2010 49 3041 3044 10.1002/anie.201000624 20309988

[63] Cui Y Tu W Floreancig PE Total synthesis of neopeltolide and analogs Tetrahedron 2010 66 4867 4873 20697460 10.1016/j.tet.2010.03.066 PMC2917233

[64] Yadav JS Kumar GG A concise stereoselective formal total synthesis of the cytotoxic macrolide (+)-Neopeltolide *via* Prins cyclization Tetrahedron 2010 66 480 487

[65] Skepper CK Quach T Molinski TF Total Synthesis of Enigmazole A from Cinachyrella enigmatica. Bidirectional Bond Constructions with an Ambident 2,4-Disubstituted Oxazole Synthon J Am Chem Soc 2010 132 10286 10292 20590095 10.1021/ja1016975

[66] Pattenden G Ashweek NJ Baker-Glenn CAG Kempson J Walker GM Yee JGK Total synthesis of (−)-ulapualide A, a novel tris-oxazole macrolide from marine nudibranchs, based on some biosynthesis speculation Org Biomol Chem 2008 6 1478 1497 18385855 10.1039/b801036f

[67] Pattenden G Ashweek NJ Baker-Glenn CAG Walker GM Yee JGK Total synthesis of (−)-ulapualide A: The danger of overdependence on NMR spectroscopy in assignment of stereochemistry Angew Chem Int Ed 2007 46 4359 4363 10.1002/anie.200700459 17465437

[68] Smith TE Kuo W Bock VD Roizen JL Balskus EP Theberge AB Total Synthesis of (−)-Hennoxazole A Org Lett 2007 9 1153 1155 17316014 10.1021/ol070244p

[69] Smith TE Kuo W Balskus EP Bock VD Roizen JL Theberge AB Carroll KA Kurihara T Wessler JD Total Synthesis of (−)-Hennoxazole A J Org Chem 2008 73 142 150 18052386 10.1021/jo7018015

[70] Nett M Erol O Kehraus S Kock M Krick A Eguereva E Neu E König GM Siphonazole, an Unusual Metabolite from *Herpetosiphon* sp Angew Chem Int Ed 2006 45 3863 3867 10.1002/anie.200504525 16671154

[71] Linder J Moody CJ The total synthesis of siphonazole, a structurally unusual bis-oxazole natural product Chem Commun 2007 1508 1509 10.1039/b618160k 17406689

[72] Linder J Blake AJ Moody CJ Total synthesis of siphonazole and its *O*-methyl derivative, structurally unusual bis-oxazole natural products Org Biomol Chem 2008 6 3908 3916 18931796 10.1039/b810855b

[73] Zhang J Polishchuk EA Chen J Ciufolini MA Development of an Oxazole Conjunctive Reagent and Application to the Total Synthesis of Siphonazoles J Org Chem 2009 74 9140 9151 19950882 10.1021/jo9018705

[74] Chandrasekhar S Sudhakar A Total Synthesis of Bengazole A Org Lett 2010 12 236 238 20017536 10.1021/ol9024138

[75] Enriquez-Garcia A Ley SV Total synthesis of the potent antifungal agents bengazole C and E *Collect Czechoslovak* Chem Commun 2009 74 887 900

[76] Bull JA Balskus EP Horan RAJ Langner M Ley SV Total synthesis of potent antifungal marine bisoxazole natural products benzazoles A and B Chem Eur J 2007 13 5515 5538 10.1002/chem.200700033 17440905

[77] Scarone L Fajardo J Saldana J Dominguez L Esposito P Demastteis S Wipf P Manta E Serra G Synthesis and evaluation of anthelmintic and cytotoxic properties of [2,5′]bis-1,3-azole analogs of bengazoles Lett Drug Des Discov 2009 6 413 419

[78] Krauss J Kalkbrenner S Schuster A Obainoke A Bracher F Synthesis of new bengazole analogues and their antimicrobial activity Turk J Chem 2008 32 125 130

[79] Mulder RJ Shafer CM Dalisay DS Molinski TF Synthesis and structure-activity relationships of bengazole A analogs Bioorg Med Chem Lett 2009 19 2928 2930 19419860 10.1016/j.bmcl.2009.04.069 PMC2691865

[80] Fresneda PM Castaneda M Blug M Molina P Iminophosphorane-based preparation of 2,5-disubstituted oxazole derivatives: synthesis of the marine alkaloid almazole C Synlett 2007 2 324 326

[81] N’Diaye I Guella G Mancini I Pietra F Almazole D, a New Type of Antibacterial 2,5-Disubstituted Oxazolic Dipeptide from a Red Alga of the Coast of Senegal Tetrahedron Lett 1996 37 3049 3050

[82] Miyake F Hashimoto M Tonsiengsom S Yakushijin K Horne DA Synthesis of 5-(3-indolyl) oxazole natural products. Structure revision of Almazole D Tetrahedron 2010 66 4888 4893

[83] Lucas BS Gopalsamuthiram V Burke SD Total synthesis of phorboxazole B Angew Chem Int Ed 2007 46 769 772 10.1002/anie.200603656 17163573

[84] Smith AB III Razler TM Meis RM Pettit GR Synthesis and Biological Evaluation of Phorboxazole Congeners Leading to the Discovery and Preparative Scale Synthesis of (+)-Chlorophorboxazole A Possessing Picomolar Human Solid Tumor Cell Growth Inhibitory Activity J Org Chem 2008 73 1201 1208 18215059 10.1021/jo701816h

[85] Smith AB III Razler TM Ciavarri JP Hirose T Ishikawa T Meis RM A Second-Generation Total Synthesis of (+)-Phorboxazole A J Org Chem 2008 73 1192 1200 18215058 10.1021/jo7018152

[86] Haberhauer G Drosdow E Oeser T Rominger F Structural investigation of westiellamide analogs Tetrahedron 2008 64 1853 1859

[87] Comba P Gahan LR Haberhauer G Hanson GR Noble CJ Seibold B van den Brenk AL Copper (II) coordination chemistry of westiellamide and its imidazole, oxazole, and thiazole analogues Chem Eur J 2008 14 4393 4403 10.1002/chem.200701778 18381720

